# Species Interactions Alter Evolutionary Responses to a Novel Environment

**DOI:** 10.1371/journal.pbio.1001330

**Published:** 2012-05-15

**Authors:** Diane Lawrence, Francesca Fiegna, Volker Behrends, Jacob G. Bundy, Albert B. Phillimore, Thomas Bell, Timothy G. Barraclough

**Affiliations:** 1 Department of Life Sciences, Imperial College London, Silwood Park Campus, Ascot, Berkshire, United Kingdom; 2 Grantham Institute for Climate Change, Imperial College London, London, United Kingdom; 3 Department of Surgery and Cancer, Faculty of Medicine, Imperial College London, London, United Kingdom; 4 Department of Zoology, University of Oxford, Oxford, United Kingdom; Cornell University, United States of America

## Abstract

Studies of evolutionary responses to novel environments typically consider single species or perhaps pairs of interacting species. However, all organisms co-occur with many other species, resulting in evolutionary dynamics that might not match those predicted using single species approaches. Recent theories predict that species interactions in diverse systems can influence how component species evolve in response to environmental change. In turn, evolution might have consequences for ecosystem functioning. We used experimental communities of five bacterial species to show that species interactions have a major impact on adaptation to a novel environment in the laboratory. Species in communities diverged in their use of resources compared with the same species in monocultures and evolved to use waste products generated by other species. This generally led to a trade-off between adaptation to the abiotic and biotic components of the environment, such that species evolving in communities had lower growth rates when assayed in the absence of other species. Based on growth assays and on nuclear magnetic resonance (NMR) spectroscopy of resource use, all species evolved more in communities than they did in monocultures. The evolutionary changes had significant repercussions for the functioning of these experimental ecosystems: communities reassembled from isolates that had evolved in polyculture were more productive than those reassembled from isolates that had evolved in monoculture. Our results show that the way in which species adapt to new environments depends critically on the biotic environment of co-occurring species. Moreover, predicting how functioning of complex ecosystems will respond to an environmental change requires knowing how species interactions will evolve.

## Abstract 2

Adaptation to a novel environment is altered by the presence of co-occurring species. Species in diverse communities evolved complementary resource use, which altered the functioning of the experimental ecosystems.

## Author Summary

Understanding how species adapt to new environments is important both for evolutionary theory and for predicting and managing ecosystem responses to changing environments. However, most research into adaptation to new environments has considered species in isolation. Whether results from these systems apply to more realistically diverse groups of species remains unclear. We exposed five species of bacteria, collected from pools around the roots of beech trees, to a novel laboratory environment either in isolation or in species mixtures for approximately 70 generations. We found that each species evolved more in diverse species mixtures than it did when cultured in isolation. Moreover, species diverged in their use of resources and how they used the waste products of other species. These changes meant that the community of bacteria that evolved together used more of the available resources and were thereby more productive than the same group of species that evolved in isolation. Our findings show that species interactions can have a major effect on evolutionary dynamics, which can in turn influence ecosystem functioning.

## Introduction

Understanding how species adapt to novel environments is an important task both for understanding the dynamics of living systems and for predicting biotic responses to anthropogenic changes in the natural environment [1]–[3]. However, most studies of evolutionary adaptation consider single species in isolation. Although this approach is useful for uncovering genetic mechanisms, virtually all species co-occur with many other species. Faced with a new abiotic environment, communities might respond by evolution of component species, but ecological changes in species' abundances and distributions can also occur. If ecological interactions such as competition affect evolutionary responses [4],[5], then results from single species studies might not accurately predict evolutionary dynamics in diverse assemblages.

Although there has been growing interest in how evolution affects ecological dynamics [6]–[10], most studies have still considered single species or pairs of interacting species. In addition, the question of how ecological interactions affect evolutionary responses to novel abiotic environments has received even less attention [11],[12]. If ecological interactions among species are weak, then evolutionary changes should be the same as those predicted in single species studies. However, if species use overlapping resources or otherwise interact, the extent and type of evolutionary responses might differ from those predicted if the same set of species each adapted to the new abiotic conditions in isolation [13],[14].

Several mechanisms might influence evolutionary dynamics in mixtures of species. First, species in diverse communities might have their resource use restricted by competitors, lowering effective population sizes and therefore reducing the rate at which beneficial mutations arise and the species adapts to a novel environment [5],[15]. In this scenario, species in communities should adapt to the new environment as they would in isolation, but the rate of adaptation would be reduced. Second, if trait variation among species exceeds variation within species, a new abiotic environment might act on the relative abundance of different species (ecological sorting) rather than on genetic variation within species [4],[5]. In models of this mechanism, pre-adapted species increase in abundance at the expense of less well-adapted species and the average amount of evolution in surviving species is typically reduced compared to responses of the same species in monoculture (although in rare scenarios the amount of evolution can increase [4]). Third, there might be a trade-off between adaptation to biotic and abiotic components of the environment [16]. Such trade-offs might result from the production of costly adaptations involved in species interactions such as defences [17],[18] or from selective interference between adaptations to the biotic and abiotic environment [19]. In this case, species that evolved in communities should be less well adapted to the abiotic environment than if they adapted in isolation and vice versa. In the most extreme case, species might adapt to use resources generated by other species [20], in which case they will evolve entirely different resource use depending on whether other species are present.

These mechanisms could change both the magnitude and direction of evolutionary change in communities compared to predictions from single species studies. However, evidence for an effect of diversity is currently scarce. Experiments have shown that diversity can inhibit evolution; for example, Brockhurst et al. [21] showed that niche occupation restricts adaptive radiation of a single bacterial strain. Similarly, Collins [16] found that diversity limits adaptation to elevated CO_2_ in algae and Perron et al. [22] showed that diversity limits the evolution of multi-drug resistance (although this effect was alleviated by horizontal transfer of resistance mutations). However, these studies considered genetic diversity within species rather than species diversity within communities. There is abundant evidence that coevolution drives fast evolution between species with strong ecological interactions [23],[24] and that pairwise coevolution can change the direction of evolution compared to adaptation in isolation [14]. Furthermore, studies of diffuse coevolution have shown that adaptation of a focal species to particular interacting species, such as insect herbivores, is influenced by interactions with other species, such as vertebrate herbivores [25]–[28]. For example, character displacement of limnetic and benthic species pairs of sticklebacks only occurred in lakes with low species diversity of other fish [29]. To the best of our knowledge, however, the evolution of interactions among multiple species in a community has not been investigated using an experimental evolution approach.

Evolutionary dynamics in diverse systems will have important consequences for ecosystem functioning in altered environments. Ecosystem functions such as decomposition and productivity emerge from the degree to which species are adapted to their biotic and abiotic environments [30]–[32]. Following a change in the environment, ecosystem functioning might be disrupted either because the species abundances change or because component species fail to adapt to the new environmental optimum. Alternatively, coevolution among species might act to enhance ecosystem properties, for example if species evolve complementary resource use and thereby increase ecosystem productivity [33]. Understanding of these processes is needed to predict how ecosystem functioning will respond to environmental changes over evolutionary timescales.

**Figure 1:**
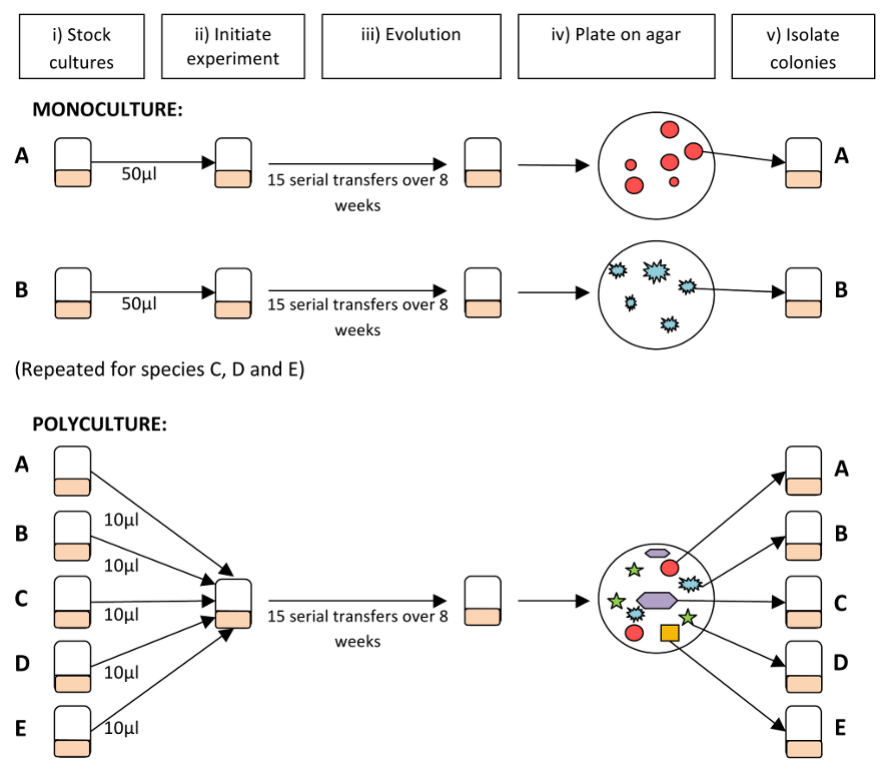
Experimental design for the evolution experiments. (i) Stocks of wild isolates were grown up, each comprising a single starting genotype of each species. (ii) Experiments were started with each species in monoculture or in polyculture (all five species mixed together). (iii) To stimulate active growth and promote adaptation to the laboratory conditions, each culture was diluted 20-fold in fresh medium twice weekly for 8 wk. Tubes were shaken to prevent the formation of biofilms and maintain spatial homogeneity. Numbers of generations ranged from 60.9 to 82.2 across cultures and effective population sizes ranged from 5.3×10^5^ to 9.9×10^6^ (Table S3). (iv) Final cultures were plated on agar. (v) Single colonies of each species were isolated for growth assays described in the main text.

Here, we test whether species diversity influences environmental adaptation and ecosystem functioning using naturally co-occurring decomposer bacteria from temporary pools around the roots of beech trees (*Fagus sylvatica*), which have previously been used successfully for experimental ecology [34],[35]. We chose five species of bacteria differing in colony colour and shape so that each species could be isolated from species mixtures (Tables S1 and S2). Sequencing of 16S rDNA showed that isolates belong to five different families (Table S1). We refer to them as species since they represent genetically and phenotypically distinct clusters that co-occurred naturally. Monocultures of each species and polycultures containing all five species were allowed to adapt to laboratory conditions by regular serial transfer on beech-leaf extract (Figure 1). Laboratory conditions represent a new environment and differ from wild tree-holes in several ways: tree-holes receive a larger quantity and variety of resources, are spatially complex, and have an unpredictable input of water and leaves, whereas laboratory cultures experienced regular dilution with uniform medium in a shaken container. Growth assays were used to determine evolutionary responses. We predicted that species should adapt to laboratory conditions by evolving faster growth rates on the beech tea medium, but that the presence of other species might change the direction and extent of adaptation by one of the mechanisms outlined above.

To measure species interactions and changes in resource use, our approach was to grow one species on beech tea, then to filter-sterilize the medium and to assay the growth of a second species on the “used” beech tea. If the second species used similar resources to the first (i.e., if their niches overlapped), the second species should grow less well on “used” beech tea than on “unused” beech tea because its resources would have been consumed. If the two species were specialized on different resources (i.e., occupied different niches), the second species should grow equally well on “used” and “unused” tea. Finally, if the second species used resources produced by the first (called facilitation or cross-feeding [36]), the second species should grow better on “used” tea than on “unused” tea. While this method does not provide direct information on competitive interactions in mixtures, it provides a tractable and reproducible measure of changes in resource use of each species during evolution. Because other types of interaction, such as direct inhibition by bacteriocides [37], might also affect growth rates, we also used nuclear magnetic resonance (NMR) spectroscopic profiling of “used” and “unused” tea to investigate changes in resource use directly.

Finally, we tested whether adaptation to the presence of other species affected productivity (rate of production of CO_2_) by reassembling communities with different evolutionary histories using isolates that either evolved in monoculture or co-evolved in the same polyculture. If adaptation increased community productivity, we expected communities reassembled with isolates that evolved in polycultures to be more productive than those reassembled with isolates that had evolved in monoculture.

## Results

### Growth Rates on Beech Tea of Monoculture Isolates

**Figure 2:**
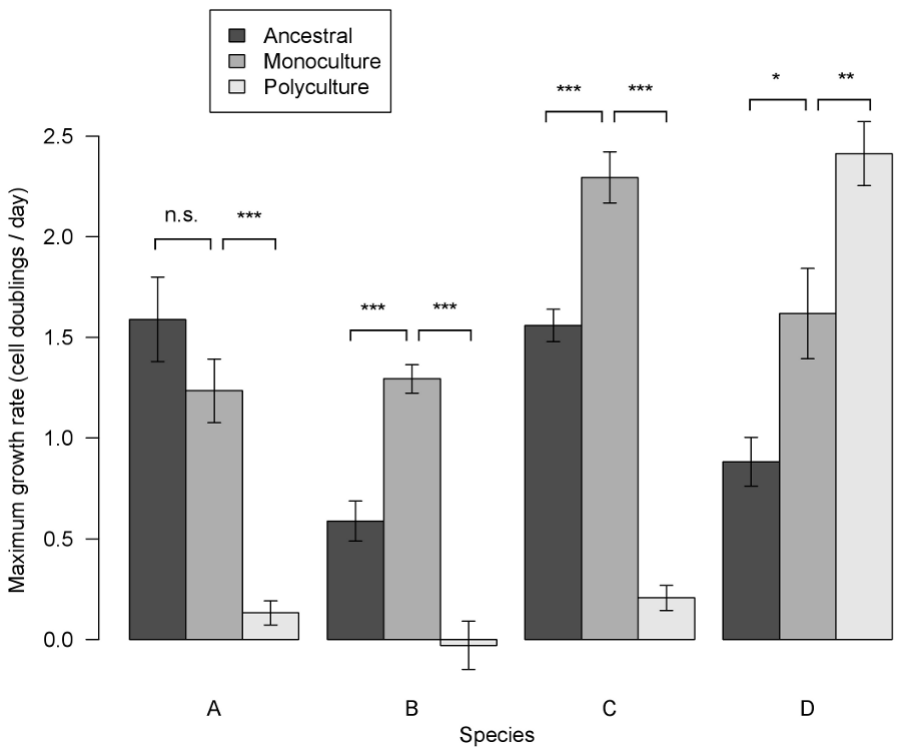
Maximum growth rates of isolates after evolution under each diversity treatment. Maximum rate of growth from low densities, *V_MAX_*, of each species grown on unused beech tea under assay conditions. Dark bars, growth rates of ancestral isolates. Mid grey bars, growth rates of monoculture isolates. Pale bars, growth rates of polyculture isolates. Standard error bars are shown. Tukey Honest Significant Difference test contrasts between treatments: *** *p*<0.001, ** *p*<0.01, * *p*<0.05; n.s., not significant (see also Table S4). Species A evolved slower maximum growth rates in polycultures compared to its ancestral and monoculture isolates. Species B and C evolved faster maximum growth rates on unused beech tea in monocultures, but far slower maximum growth rates in polycultures compared to ancestral isolates. Species D evolved faster maximum growth rates in monocultures compared to its ancestral isolate and even faster maximum growth rates in polycultures.

Although able to grow on beech tea in the lab at the start of the experiment, one species (E) dwindled to low cell densities during the evolution experiment (Table S3) and was excluded from growth assays and subsequent experiments because it failed to re-grow from frozen cultures. Species A to D were recoverable in all treatments and were used for subsequent experiments. Across species, final isolates that evolved in monoculture grew on average faster than ancestral isolates of the same species on unused beech tea (dark bars, first and second rows, Figure 2), consistent with the prediction that they adapted to laboratory conditions of serial dilution in beech tea medium by increasing growth rates on this medium. The effect was significant in species B, C and D, which grew between 47% and 120% faster after evolving in monoculture compared to their ancestral isolates. Growth rates of evolved monoculture isolates of species A were not significantly different from its ancestral isolate. Note that phenotypic plasticity and parental effects can be discounted as explanations for differences among treatments. In all our assays, frozen isolates were first grown in beech tea medium for 4 d ( = 4 to 6 generations, Table S3), and then an aliquot was taken from these cultures to start the assay cultures. Differences in phenotypes between treatments were therefore maintained after several generations of growth in identical environments and cannot be readily explained by phenotypically plastic responses.

### Growth Rates on Beech Tea of Polyculture Isolates

Isolates of species A, B, and C that evolved in polyculture grew significantly slower on unused beech tea than their corresponding ancestral isolates and than the isolates that evolved in monoculture (Figure 2). Growth rates were 87% to 100% slower after evolving in polyculture compared to the corresponding ancestral isolates. This is consistent with the existence of a trade-off between adaptation in the presence of other species and adaptation in the absence of other species; when evolving in the presence of other species, isolates of A, B, and C nearly lost the ability to grow on unused beech tea. In contrast, the polyculture isolate of species D grew significantly faster on unused beech tea than either ancestral or monoculture isolates. By adapting in the presence of the other species, species D evolved to grow at a faster rate on beech tea when assayed with other species absent. This result is not readily predicted by the general theories outlined in the introduction and is discussed further below.

### Species Interactions between Ancestors and between Isolates Evolved in Monoculture

**Figure 3:**
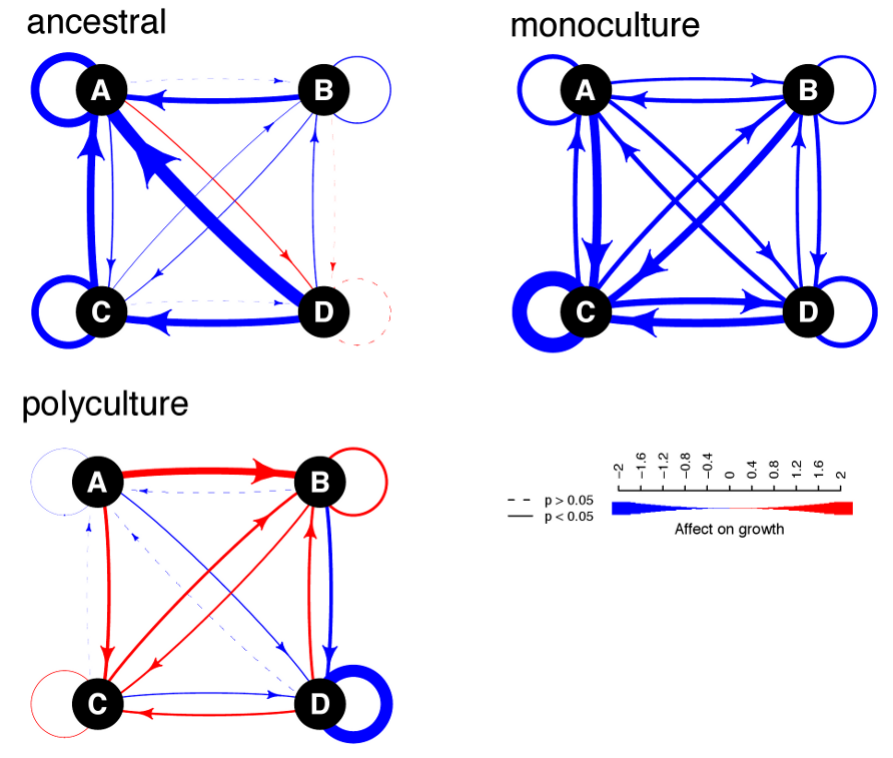
The interspecific impacts of resource use on relative growth. Interspecific effect on relative growth among species inferred from their ability to grow on sterile beech tea previously used by each other species, shown separately for each treatment. Blue arrows indicate negative effects on growth, and red arrows indicate positive effects on growth. The width of the arrow represents the maximum growth rate (*V_MAX_*) on used tea minus the maximum growth rate on unused tea (underlying data in Figure S1 and linear model in Table S4). Dashed lines indicate that growth on used tea was not significantly different from growth on unused tea.

Reduced growth of ancestral isolates on beech tea previously used by other ancestral isolates showed that species had generally negative interactions (Figures 3A, S1), as predicted if species used overlapping resources. The exception was species D, whose growth was not reduced on tea previously used by other species even though tea used by species D reduced the growth of other species (arrows towards species D on Figure 3A). This result might indicate that species D used a greater range of resources than the other species, but which included the resources used by the other species. Growth of monoculture isolates on tea previously used by monoculture isolates of other species showed that negative interactions among species were reinforced: now species D also grew significantly slower on tea previously used by other species (Figure 3B). These results would be expected if species in monoculture converged to use a more similar set of resources.

### Species Interactions between Isolates that Evolved in Polycultures

Species interactions evolved to be more positive between polyculture isolates than between ancestral or monoculture isolates (Figure 3C). Species B and C evolved in polyculture to grow significantly faster on tea previously used by other species than on unused beech tea (Figures 3C and S1). Thus, interactions shifted to facilitation as predicted if species adapted to use resources being produced by other species as waste products of metabolism. Polyculture isolates of D remained negatively affected on substrate used by other species, although less so than their monoculture isolates (Figure S1, relative growth rate on used tea versus unused tea: in monoculture, 0.24±0.05, and in polyculture, 0.77±0.07), indicating that species D also adapted to the presence of other species. Polyculture isolates of species A grew poorly on all substrates (Figure S1), but again the interactions were significantly less negative than between ancestral and between monoculture isolates.

### Resource Use of Ancestral, Monoculture, and Polyculture Isolates

**Figure 4:**
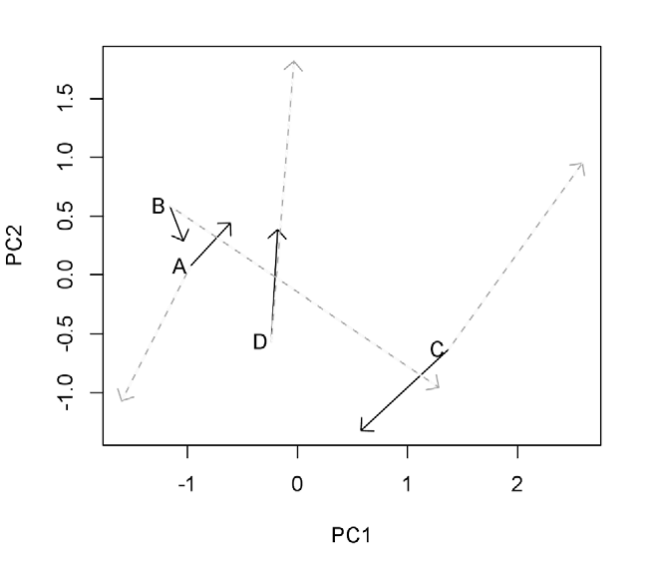
Evolution of resource use. Trajectories of evolution in monoculture (solid black arrows) and polyculture (grey dashed arrows) of each species with respect to the first two principal components summarizing variation in their ability to use and produce compounds identified by NMR. The start of each arrow indicates the position of the ancestral isolates along these axes. Increasing PC1 is correlated with using more glucose, choline, formate and succinate, and producing more pyruvate (Figure S4). Increasing PC2 is correlated with using more acetate, formate, and producing more lactate. Species resource use evolved more in polyculture than in monoculture (dashed grey arrows are longer than solid black ones), and polyculture isolates display greater divergence in resource use and production than either ancestral or monoculture isolates (dashed grey arrows point towards the four corners of the plot).

Forty-three separate resonances (i.e., peaks) were distinguished and integrated from the NMR spectra (Figures S2 and S3). Variation in the net use and production of peaks in the spectra across ancestral, monoculture, and polyculture isolates of each species confirmed that resource use evolved in each of the species in ways that matched findings from the growth assays (Figures 4, S2, and S3). Considering the multivariate space of resource use and production across all compounds, polyculture isolates displayed greater differences from ancestral isolates than did monoculture isolates (across species, mean and standard error of Euclidean distance between paired ancestral isolates and monoculture isolates = 1.20±0.26; mean and standard error of distance between ancestral isolates and polyculture isolates = 2.42±0.37, *p* = 0.003, Monte Carlo simulation). Moreover, although species evolved, if anything, to have marginally more similar resource use in monoculture (not significantly so, *p* = 0.36, Monte Carlo simulation), patterns of resource use and production diverged significantly between species in polycultures (*p* = 0.010, Monte Carlo simulation; mean and standard error of Euclidean distance between species: ancestral isolates = 2.27±0.01; monoculture isolates = 1.98±0.01; polyculture isolates = 3.41±0.01). Together these results show that species' use of NMR-visible carbon substrates in the beech tea evolved more in polyculture treatments than in monoculture treatments and did so in a way to increase the differences in metabolism between species and thereby to reduce negative interactions between them.

Principal components analysis identified the main axes of variation in net use or production of these compounds across ancestral, monoculture, and polyculture isolates of each species (Figure 4). The first principal component distinguished isolates based on the degree to which they used glucose, choline, formate, and succinate to produce pyruvate (Figure S4). The second principal component distinguished isolates based on whether they used up or produced acetate, formate, and lactate. Notable changes in polyculture isolates were as follows: species A evolved to produce 96% more acetate and to produce rather than use formate; species B evolved to use up to 84% more choline, formate, and lactate and to use rather than produce succinate; species C evolved to use rather than produce acetate; and species D evolved to produce rather than to use lactate and to use rather than produce acetate (Figure S3).

**Figure 5:**
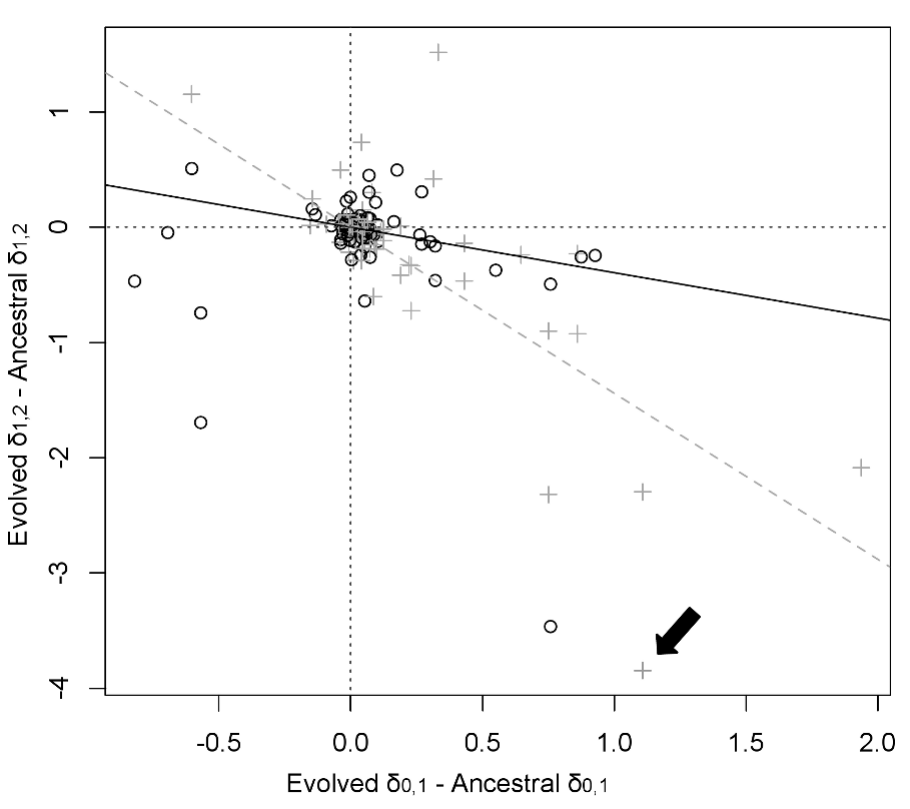
Correspondence between compounds being generated and compounds being used up by other species in polycultures. The data summarize results from assays growing one species on beech tea medium, filtering that medium, and then growing a second species on the used medium. We calculated two quantities: δ_0,1_ = the amount of compound in the filtrate from species 1 minus the amount of compound in beech tea (relative to the amount of the DSS standard); δ_1,2_ = the amount of compound in the filtrate from species 2 minus the amount of compound in filtrate from species 1. Positive δ indicates production of compounds during the assay and negative δ indicates consumption. We then compared δ between evolved and ancestral isolates for different species pairs: each point shows the comparison for a given species pair and either monoculture (black circles) or polyculture (grey crosses) treatments. The *x*-axis is δ_0,1_ of the evolved isolate minus δ_0,1_ of the corresponding ancestral isolate. More positive values indicate that the evolved isolate of the first species produced more of that compound than did its ancestral isolate. To focus on waste products as potential targets of cross-feeding, only compounds that were produced by the evolved isolate were included. The *y*-axis is δ_1,2_ for evolved isolate minus δ_1,2_ for the corresponding ancestral isolate. More negative values indicate that the evolved isolate of the second species used more of the compound than did its ancestral isolate. For example, the point indicated by the arrow represents increased production of acetate by species A in polyculture relative to ancestral isolates (*x*-axis) and its increased use by species D in polyculture relative to ancestral isolates (*y*-axis, all changes shown separately by species and compound in Figure S5). There is a general negative trend: if the first species produces more of a compound, the second species is likely to use more of it. However, the effect is significantly stronger in polyculture isolates (grey dashed line) than in monocultures (black line): polyculture isolates have evolved increased consumption of compounds that have increased in production in polyculture isolates of other species. Linear model of y = x * treatment (monoculture or polyculture), interaction term coefficient = −1.13, *t* = −5.4, *p*<0.0001.

These observed changes indicate possible cases of cross-feeding evolving in polycultures, which might explain the positive interactions observed in growth assays. For example, species D evolved to produce lactate in polycultures and species B to use it. To test whether species generally evolved increased use of other species' waste products in polycultures, we quantified the amounts of substrates produced by each species grown on beech tea and the amounts of the same substrates that were used by a subsequent species grown on the “used” beech tea (Figure S5). On average across species, polyculture isolates displayed significantly increased use of substrates (i.e., a more negative change in the amounts of the substrate on the *y*-axis of Figure 5) that were produced in increased amounts by other species (a more positive change in the amount of substrates on the *x*-axis of Figure 5, Pearson's correlation, *r* = −0.74, *p*<0.0001), relative to ancestral isolates. Moreover, although monoculture isolates were also able to use waste products generated by other monoculture isolates, the correlation between increased production and increased use (relative to ancestral isolates) was significantly weaker (Pearson's correlation *r* = −0.20, *p* = 0.03; significant interaction between slope and treatment, linear model results in Figure 5). Polyculture isolates therefore appear to have evolved greater use of waste products generated by polyculture isolates of other species.

### Ecosystem Functioning

**Figure 6:**
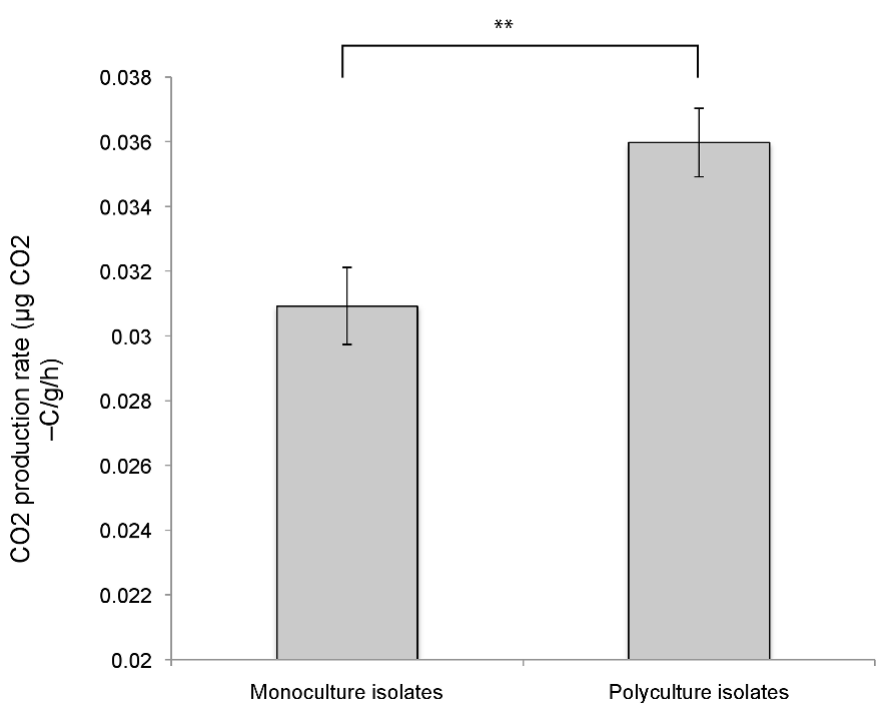
The effect of diversity during evolution on ecosystem function of reassembled communities. The mean rate of CO_2_ release over a 6-h period by communities reassembled from isolates that evolved under the two diversity treatments. Standard errors are shown. Tukey Honest Significant Difference test contrasts between interaction types: ** *p*<0.01.

Communities were reassembled to contain one isolate of each of the four surviving species. Communities reassembled using isolates that evolved in polycultures displayed significantly higher productivity, measured as CO_2_ production rate, than communities reassembled using isolates that evolved in monoculture (Figure 6). Adaptation to the biotic environment of co-occurring species therefore increased community productivity.

## Discussion

Our results show that species interactions had a major impact on how species adapted to the new environment in the laboratory. In all four surviving species, the magnitude of evolution in terms of changes in growth rate on beech tea medium and changes in use of NMR-visible resources was significantly greater in polycultures than in monocultures. Moreover, species diverged in resource use in polycultures compared to monocultures and ancestral isolates. This provides experimental evidence for a classic scenario of character displacement reducing the overlap of resources used by interacting species [38]. Furthermore, not only were negative interactions reduced, but species also adapted to use waste products of other species in polycultures, leading to positive interactions between some pairs of species. Together, these changes led to increased productivity of the entire community. By evolving to use different resources, and to metabolise waste products of other species, the species collectively decomposed substrates in the beech tea more effectively. Similar results have been observed for cross-feeding ecotypes evolving during monoculture experiments [33],[39]; here we show that cross-feeding also evolves readily between distantly related species of bacteria.

The effect of species interactions on evolution varied among species. In three species, A, B, and C, there was a trade-off between adaptation to the laboratory environment in the presence of other species and adaptation in the absence of other species: polyculture isolates grew less well when assayed in isolation than did monoculture isolates. In species B and C this occurred because they adapted to use waste products generated by other species, which was demonstrated both by their increased growth on medium previously used by other species and by their increased use of waste products from other species. In species A it occurred because this species changed to use different carbon sources than the other species: its interactions became less negative in the polyculture treatment than between ancestral or monoculture isolates (but not positive) and it used more trehalose and less glucose and lactate (Figure S3).

In contrast, species D displayed a positive effect of diversity on its adaptation to abiotic conditions: the polyculture isolate had enhanced growth rate when assayed on its own compared to either the ancestral or monoculture isolates. There is no evidence that species D polyculture isolates evolved to use any of the NMR-visible resources more effectively than any other isolates. We therefore hypothesize that polyculture isolates of species D evolved increased use of complex carbon sources that cannot be distinguished by NMR. One clue supporting this hypothesis is that polyculture D produced large amounts of lactate and was the only isolate to do so and without correlated negative change in any other compound. We suggest therefore that species D could be producing lactate from metabolism of compounds not distinguishable by NMR—for example, macromolecular structures such as mixtures of proteins. None of the general theories outlined in the introduction readily explain why species D should enhance its ability to grow on its own after evolving in polyculture. However, in rare circumstances in the niche simulation model by de Mazancourt et al. [4], competition among species could “push” one species to evolve into a wider range of niches than it would do so when in the absence of competitors. The observation that species D has shifted away from its ancestral and monoculture isolates in resource use and away from the polyculture isolates of other species is consistent with this possibility (Figure 4).

Despite differences in response among the species, in all cases the effects of diversity arose because co-adaptation between species altered their ability to grow in an environment free of other species. The other mechanisms outlined in the introduction cannot explain our results. Effective population sizes were generally lower in polycultures (Table S3), but still exceeded 10^6^ in all surviving species, and polyculture isolates did not adapt more slowly than monocultures. Instead, co-adaptation with other species rendered species A, B, and C even less well adapted to the abiotic environment in the absence of other species than their ancestors, and species D better adapted. Similarly, our results do not reflect the damping of evolutionary responses by ecological sorting, because species' use of NMR-visible compounds in fact evolved more in polycultures than in monocultures. Species E might have dwindled to low numbers in polycultures because of one of these two mechanisms (Table S3), but in any case it failed to sustain large populations during the experiment even in monocultures.

The NMR results show that changes in resource use can explain observed changes in interactions and productivity (see also [40]). It remains possible that other interactions could be operating among these species as well, but which remained undetected by our assays. Some of the metabolites generated by species could have had toxic effects on other isolates, and some of the observed metabolic changes could have been to reduce toxic effects rather than increase resource use. Also, bacteria are known to produce signalling molecules that can have inter-specific effects—for example, antimicrobial properties [37] or positive effects such as stimulating enzyme production [41]. In principle, these could have caused some of the changes in growth rates we observed in interaction assays and they would be interesting traits to investigate in future studies. However, typical signalling molecules such as quorum sensing compounds are usually not produced at high enough concentrations for detection by NMR [42], and therefore the changes observed here reflect changes in resource use rather than changes in signalling. Because the NMR results match inferences from the growth assay results, it is more parsimonious to conclude that changing resource use is the dominant mechanism explaining our findings.

Our results provide among the first experimental evidence supporting recent theories that species interactions in diverse communities affect evolutionary responses to an environmental change. The way in which species adapted to new conditions in the laboratory when in monoculture—the setting assumed for many evolutionary theories and experiments—provided little information on the outcome of evolution in the diverse community. Co-occurring species modified the environment by generating new resources, and thereby altered the selection pressures on other species and how they used the available resources. Other experiments have reported that genetic diversity inhibited adaptation to the environment [16],[21] but have not investigated whether adaptation to the biotic environment of co-occurring species changed how species adapt to a new abiotic environment. If the processes we observed here are common in other communities, including multicellular eukaryotes over longer timescales, then attempts to understand evolutionary dynamics in the wild must take into account the biotic environment of co-occurring species [13],[43].

As well as being important for understanding evolutionary dynamics, our experiments show that evolutionary interactions had important consequences for ecosystem-level functions. Co-adaptation for approximately 70 generations—not an unrealistic timescale for responses of annual eukaryotic organisms to predicted changes over the next hundred years—acted to enhance community productivity, through the evolution of complementary use of resources. Niche complementarity and facilitation are known to be important determinants of community productivity [44],[45], and our results add to growing evidence from microbial systems that niche evolution can exert a strong influence on productivity [10],[46]. Recent work has shown that biofilms derived from a single clone of *Burkholderia cenocepacia* evolved cross-feeding morphotypes that together had enhanced productivity compared to the morphotypes grown alone [33]: our study demonstrates similar processes operating between phylogenetically distinct species. It remains to be determined whether adaptation generally acts to enhance ecosystem productivity [47],[48], but if so, it will be an important process to consider in predicting the impacts of current environmental changes on ecosystem services. Ecosystem functions such as decomposition rate might be reduced by local extinction of species providing important functions, but it is important to know whether evolution of surviving species will restore (as found here) or further disrupt those functions.

Our communities were far less diverse and far simpler than natural communities. A single tree-hole likely contains thousands of bacterial species, including anaerobes and many other functional groups excluded by our isolation protocol. Even the comparatively depauperate community of multicellular eukaryotes in tree-holes would typically contain many more than four or five species [49]. A major goal for future research is to understand whether our findings scale to natural ecosystems and how other ecological mechanisms such as predation affect evolutionary outcomes in diverse communities. Strong interactions have been demonstrated between bacteria and their phages in natural settings [50], but reciprocal co-adaptation between bacterial species might be rare compared to adapting to the general biotic environment because of the large number of potential pairwise interactions among species [51]. Another important process in natural communities is immigration, which can add variants (new genotypes or species) that might swamp evolutionary responses [52]. Understanding how natural assemblages respond to new environments, such as those caused by global warming, ocean acidification, or pollution, depends critically on understanding the balance between ecological and evolutionary responses of the kind we demonstrate here.

## Materials and Methods

### Species and Media

Bacteria were isolated from single colonies from temporary pools formed by the roots of a beech tree at Silwood Park, Berkshire, United Kingdom, in November 2008 (Text S1). BLAST and Ribosomal Database Project [53] matches and photographs of colonies of each species are provided in Tables S1 and S2. Species A and E belong to families Sphingobacteriaceae and Flavobacteriaceae, respectively (both in the phylum Bacteroidetes); species B and C belong to families Enterobacteriaceae and Pseudomonadaceae, respectively (both in the class Gammaproteobacteria of the phylum Proteobacteria); and species D belongs to the family Sphingomonadaceae (in the class Alphaproteobacteria of the phylum Proteobacteria). Note that our isolation protocol means that all our bacteria are expected to be aerobic heterotrophs. Isolates were grown on beech-leaf tea prepared by autoclaving 50 g of autumn fall beech leaves in 500 ml of water and diluting the filtrate 32-fold [34].

### Evolution Experiment

Fifteen replicates of each species in monoculture and of each five-species community were set up following the protocol in Figure 1 and Text S1. The tubes were incubated at 25°C and shaken at 100 rpm. Every 3 and 4 d, 100 µl from each microcosm was transferred to 2 ml of fresh media for a total of 15 serial dilutions over 8 wk. Cell densities prior to transfer were estimated by colony counts on R2A agar. Bacteria were isolated from final cultures by plating on R2A agar, selecting single colonies, and re-suspending them in 1 ml of 1/32× beech tea. Isolates were stored at −84°C for use in subsequent assays.

### Growth Assays on Unused and Used Beech Tea

Growth assays were performed in 1 ml of 32× beech tea in 24-well plates inoculated with 250 µl of bacteria from a liquid culture grown up for 4 d from stored frozen isolates. The plates were kept at 25°C for 4 d without shaking and growth measured daily using OD_600_. Readings were subtracted from negative controls of sterile medium placed on each column of the plate. Nine replicates were used for each Species×Treatment combination. “Used” beech tea was prepared by inoculating 14 ml of beech tea with 200 µl of single bacterial species and allowing growth at 25°C for 14 d. The first and second isolate used for each assay always belonged to the same treatment—that is, both ancestral, both monoculture, or both polyculture isolates. Substrates were then filter sterilized using a 0.2 µm membrane to remove bacterial cells and leave any unused nutrients in the substrate. Sterility was confirmed by plating on agar. Growth was measured as described for growth assays on unused beech tea for nine replicates of each Species×Substrate×Treatment combination.

### Nuclear Magnetic Resonance (NMR) Analyses

Samples of unused beech tea, tea used previously by one isolate, and tea used previously by one isolate and then a second isolate (as described in the previous section) were analysed using proton NMR. Because of the low concentration of carbon substrates in the beech tea, 5 ml of each sample were lyophilized and resuspended in 550 µl 90% ^2^H_2_O (superscript numbers are atomic weights; i.e., ^1^H_2_O is “normal” water and ^2^H_2_O is deuterated) containing 1 mmol l^−1^ 3-(trimethylsilyl)propane-1-sulfonic acid (DSS) and, 5 mmol l^−1^ sodium azide. The ^2^H_2_O provided a field frequency lock for the spectrometer and the DSS served as an internal chemical shift reference. Spectra were acquired on a Bruker 800 US^2^ NMR spectrometer (Bruker BioSpin), with a magnetic field strength of 18.8 T and resulting ^1^H resonance frequency of 800 MHz, equipped with a 5-mm cryogenic probe. Spectra were acquired following the approach given in [54]. Briefly, a one-dimensional NOESY pulse sequence was used for water suppression; data were acquired into 64 k data points over a spectral width of 12 kHz, with eight dummy scans and 256 scans per sample. Spectra were phased in iNMR 3.6 (Mestrelab) and exported to Matlab 2010b (Mathworks) for further analysis. Distinct peaks were integrated and baseline-corrected using in-house scripts and assigned where possible using in-house databases. One resonance with a singlet at chemical shift δ = 3.22 ppm was assigned as choline; a COSY spectrum of the unused medium showed a cross-peak at δ 4.05/3.52 ppm, as would be expected for the methylene protons of choline (although the resonances were too low intensity to be visible in the 1D spectra). To measure resource use or production, we calculated the size of each peak in medium obtained after the growth of an isolate minus the size of the peak in the medium before the species had grown on it. Positive values indicate net production of a compound and negative values indicate net consumption. We used correlation tests to identify correlated peaks with *r*>0.95, which might indicate multiple peaks derived from the same compound. Contaminant peaks derived from methanol and acetonitrile were removed from the dataset. Variation in resource use and production across isolates was explored using principal components analysis of unscaled variances implemented with the prcomp() function in R [55]: we used unscaled rather than scaled variances to focus on compounds showing larger changes in their absolute concentrations.

### Productivity of Assembled Communities

MicroResp kits were used to measure community respiration. Respired CO_2_ results in a change in colour of cresol red indicator dye suspended above each well of a 96-well plate. Ten replicates were used per treatment in a single plate and the experiment was repeated in triplicate. Each well contained 840 µl of 1/32× beech tea and 40 µl of each species from a stock culture of standard density. The plate was sealed and the change in optical density (OD) at 570 nm of the indicator gel measured after 6 h as recommended by the manufacturers [56]. The change in OD of blank wells (filled with 1 ml 1/32× beech tea) was used to account for the base level of CO_2_ in the vials. The rate of CO_2_ respiration per ml of culture medium was calculated using the formula provided in the MicroResp manual [56].

### Statistical Analysis

To calibrate OD_600_ in terms of cell density per ml of culture medium [57], we performed serial dilution and colony counts of stock cultures of isolates of each species from each treatment. We fitted a linear model with log (colony count)/ml as the response variable and species, treatment, and OD_600_ as explanatory variables, including interaction terms. The model simplified to retain species and OD_600_, but no interaction terms (i.e., different intercept for calibration line for each species, but same slopes, *F*_4,67_ = 32.9, *p*<0.0001, *r* ^2^ = 0.64, Figure S8). The fitted lines were used to calibrate in units of log(number of cells) per ml. We used linear mixed effects models of repeated measures of cell density over time to compare growth of bacteria among treatments and species in the growth assays (Text S1). To report the direction and effect size of differences among treatments, we used the rate of change in density over the first 48 h as a simple measure of *V_MAX_*—that is, the maximum rate of growth from low densities (Figure S6). Analysis of variance (ANOVA) and Tukey's Honest Significant Difference tests were used to identify significant contrasts between particular treatments of interest. There was no evidence of different evolutionary trends in carrying capacity of isolates (i.e., using density at 96 h) as opposed to growth rate (Figure S1 versus Figure S7). To test for significant differences in NMR profiles between treatments, we used Monte Carlo simulation tests shuffling profiles randomly among species and treatments. The Euclidean distance between samples was recorded, and the mean distance between both evolved treatments in turn and ancestral isolates was used to measure the amount of evolution, and the mean distance between each species within a treatment was used to measure the amount of divergence in resource use among species. Observed values were compared to randomised values from 10,000 random permutations. Two-tailed tests were used.

## Supporting Information

Figure S1 Maximum growth rates for each species and evolution treatment when grown in “used” and “unused” substrate. Boxplots of maximum growth rates, *V_MAX_*, in cell doublings per day across evolution treatments, species, and substrates. The dark line shows the median, the box limits show the inter-quartile range, and whiskers/points indicate extreme values.
(TIF)

Figure S2 Amounts of compounds identified from distinct peaks in the NMR spectrum of unused beech tea. Bars show the size of the major peak for each distinct compound relative to the size of the standard, DSS; hence peak heights are dimensionless. The location of each peak on the spectrum is shown after each name (peak shift in parts per million).
(TIF)

Figure S3 NMR peaks for each species and treatment. The difference in the size of NMR peaks between tea used by ancestral (dark grey), monoculture (mid grey), and polyculture (light grey) in turn and the size of peaks in unused beech tea. Positive values indicate production of a compound, and negative values indicate consumption of a compound. Peak sizes are expressed relative to the size of the standard, DSS, and hence are dimensionless.
(TIF)

Figure S4 Contribution of each compound to variation between treatments. Loadings of the first four principal components of resource use and production of the four surviving species across ancestral, monoculture, and polyculture treatments. The input data were the difference between the size of the peak in medium used by the isolate and the size of the peak in the beech tea (i.e., the data in Figure S3). Bars indicate the correlation coefficient between variation in each compound and the relevant principal component. The percentage of total variation described by each principal component is shown above each plot; together they explain 90.1% of the total variation.
(TIF)

Figure S5 Changes in substrate composition after use by a first species and then species B or D. The difference in the relative size of NMR peaks between tea used by a first species' ancestral (red), monoculture (green), and polyculture (blue) in turn and the relative size of peaks in unused beech tea; together with the change in the size of the peak after a second species grew on medium already used by the first species (then filter sterilised) for the same treatments (ancestral, pink; monoculture, light green; polyculture, light blue). The order of bars for each compound is first species ancestral, second species ancestral, first species monoculture, second species monoculture, first species polyculture, and second species polyculture. Positive values indicate production of a compound, and negative values indicate consumption of a compound relative to the starting medium. To improve clarity of the figure and focus on compounds of interest for cross-feeding, only compounds in which at least one isolate generated an increase in peak size of 0.5 are shown. Only species B and species D were used as the second species, chosen to represent two species showing different results in the growth assays. Evidence of evolved cross-feeding in polyculture is apparent when high blue peaks (generation of the compound by the first species) are associated with low purple peaks (use of the compound by the second species). For example, the species A polyculture isolate produces formate, which in turn is used up by both species B and D.
(TIF)

Figure S6 Growth of replicates of each species in assays on unused beech tea across the three treatments. The *y*-axes are log(cell counts per ml), and *x*-axes are time since start in hours. Ancestral isolates of all four species grew linearly over the assay period on unused beech tea (ANOVA comparing a model with time as a factor versus a model with time as a continuous variable, likelihood ratio = 6.9, *df* = 13 and 21, *p* = 0.55). The monoculture isolates displayed significantly non-linear growth (ANOVA comparing models with time as a factor and as a continuous variable, L-ratio 39.3, *p*<0.0001). In species A, B, and C there was a reduction in growth rate between day 2 and 3 followed by recovery by day 4. In species D, there was a successive decline in growth rate. In each case, growth between day 0 and day 2 was faster than at any later period. Polyculture isolates grew linearly over the assay period (ANOVA comparing models with time as a factor and as a continuous variable, L-ratio 27.7, *p*<0.001).
(TIF)

Figure S7 Boxplots of the density after 4 d (log10) across species and substrates. The dark line shows the median, the box limits show the inter-quartile range, and whiskers/points indicate extreme values. Key findings based on comparing Vmax remain the same when comparing amount of growth by day 4: species A grows well on unused tea in ancestral and monoculture treatments, but not when it has evolved in polyculture. Species B and C shift from having reduced growth on used tea in ancestral and monoculture isolates to having enhanced growth in polyculture treatments. Species D evolves to have stronger negative effects of used tea in monoculture than in ancestral isolates, but evolves even better growth on unused tea when it evolves in polycultures than in either ancestral or monoculture isolates.
(TIF)

Figure S8 Scatter plot showing the linear relationship between OD_600_ and log colony counts. The model simplified to retain species and OD_600_, but no interaction terms (i.e., different intercept for calibration line for each species, but same slopes, *F*_4,67_ = 32.9, *p*<0.0001, *r* ^2^ = 0.64). The fitted lines were used to calibrate in units of log(number of cells) per ml.
(TIF)

Table S1 Molecular identification of bacterial isolates.
(DOCX)

Table S2 Description and photographs of growth morphology of each species on agar plates.
(DOCX)

Table S3 Densities, doubling rates, and effective population sizes of each species during the evolution experiments.
(DOCX)

Table S4 Linear mixed effects model comparisons.
(DOCX)

Text S1 Additional methods and references.
(DOC)

## Abbreviations

ANOVA: Analysis of variance
NMR: nuclear magnetic resonance
OD: optical density
