# Supplementary figures and images for: Species Interactions Alter Evolutionary Responses to a Novel Environment
Source: PLoS Biol. 2012 May 15;10(5):e1001330. doi: 10.1371/journal.pbio.1001330 (PMC3352820; doi:10.1371/journal.pbio.1001330)

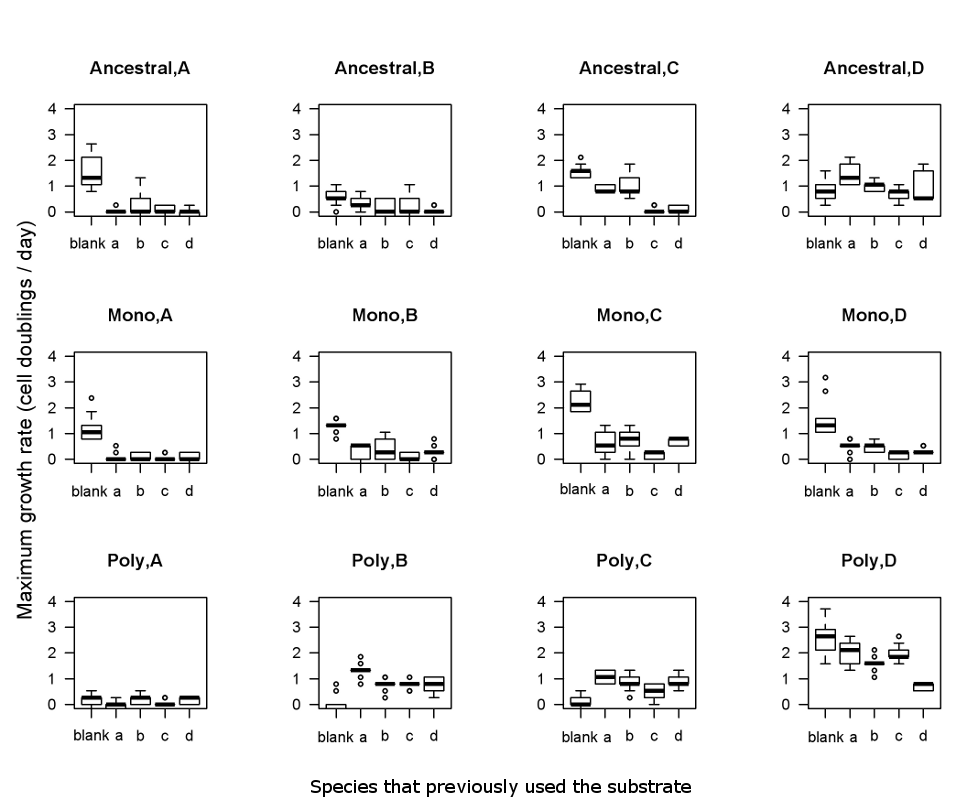

Supplement: Figure S1 — Maximum growth rates for each species and evolution treatment when grown in “used” and “unused” substrate. Boxplots of maximum growth rates, VMAX, in cell doublings per day across evolution treatments, species, and substrates. The dark line shows the median, the box limits show the inter-quartile range, and whiskers/points indicate extreme values. (TIF) [file pbio.1001330.s001.tif]

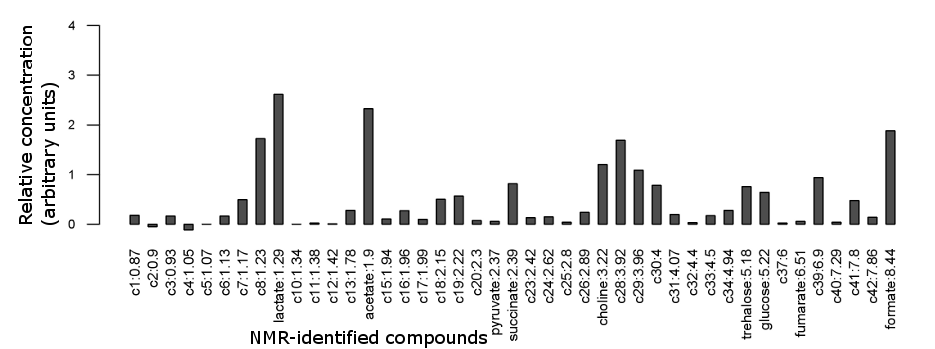

Supplement: Figure S2 — Amounts of compounds identified from distinct peaks in the NMR spectrum of unused beech tea. Bars show the size of the major peak for each distinct compound relative to the size of the standard, DSS; hence peak heights are dimensionless. The location of each peak on the spectrum is shown after each name (peak shift in parts per million). (TIF) [file pbio.1001330.s002.tif]

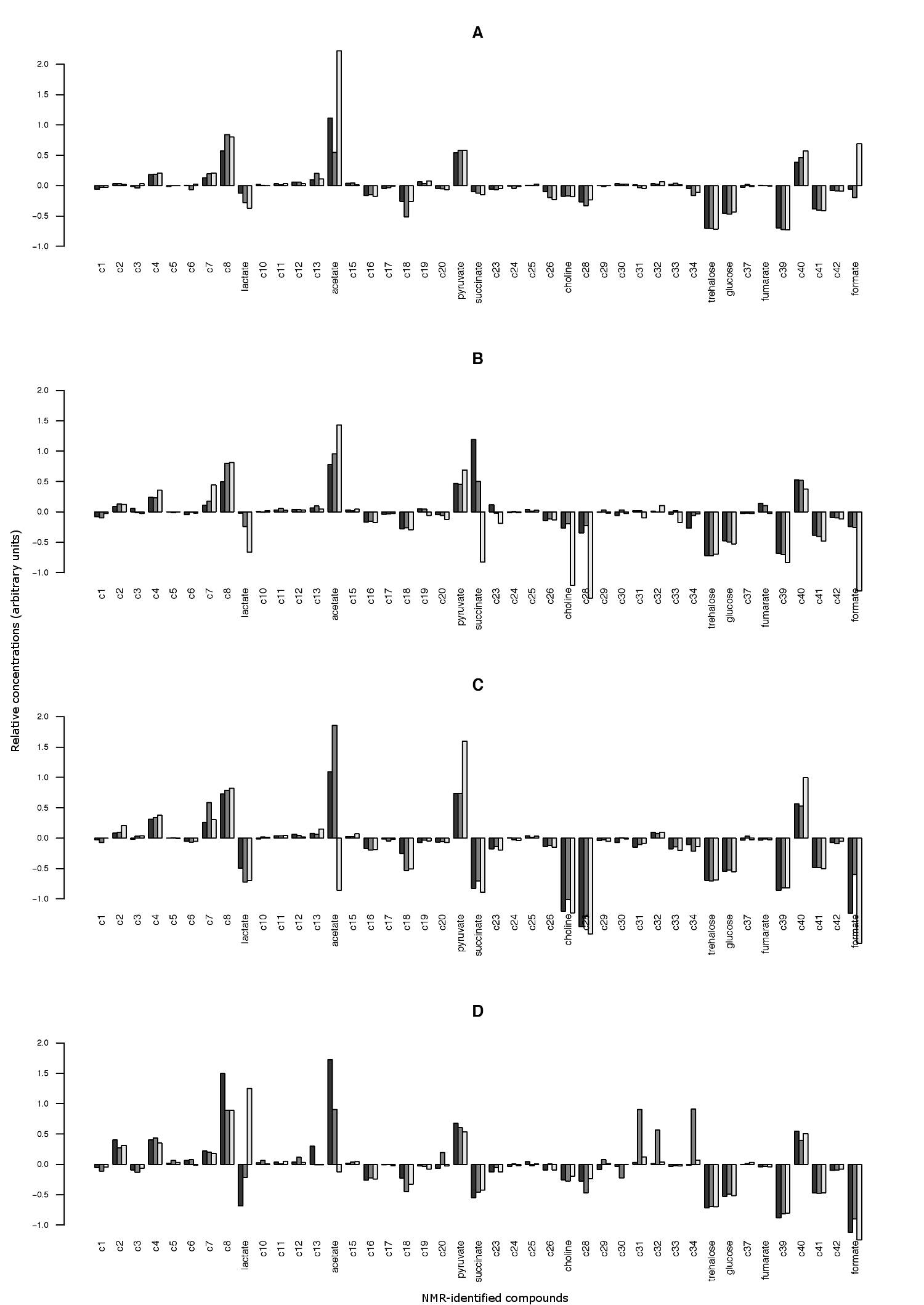

Supplement: Figure S3 — NMR peaks for each species and treatment. The difference in the size of NMR peaks between tea used by ancestral (dark grey), monoculture (mid grey), and polyculture (light grey) in turn and the size of peaks in unused beech tea. Positive values indicate production of a compound, and negative values indicate consumption of a compound. Peak sizes are expressed relative to the size of the standard, DSS, and hence are dimensionless. (TIF) [file pbio.1001330.s003.tif]

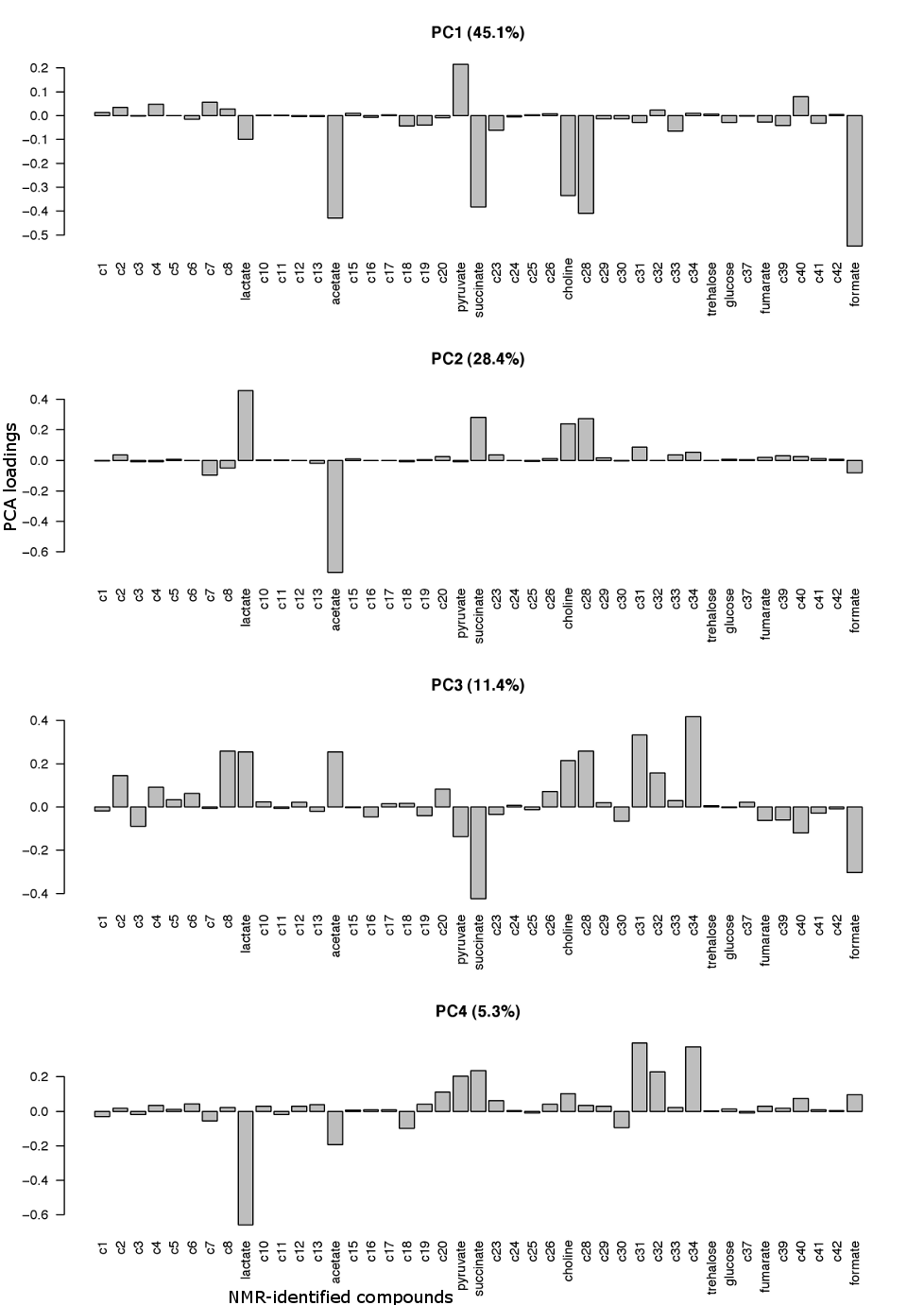

Supplement: Figure S4 — Contribution of each compound to variation between treatments. Loadings of the first four principal components of resource use and production of the four surviving species across ancestral, monoculture, and polyculture treatments. The input data were the difference between the size of the peak in medium used by the isolate and the size of the peak in the beech tea (i.e., the data in Figure S3). Bars indicate the correlation coefficient between variation in each compound and the relevant principal component. The percentage of total variation described by each principal component is shown above each plot; together they explain 90.1% of the total variation. (TIF) [file pbio.1001330.s004.tif]

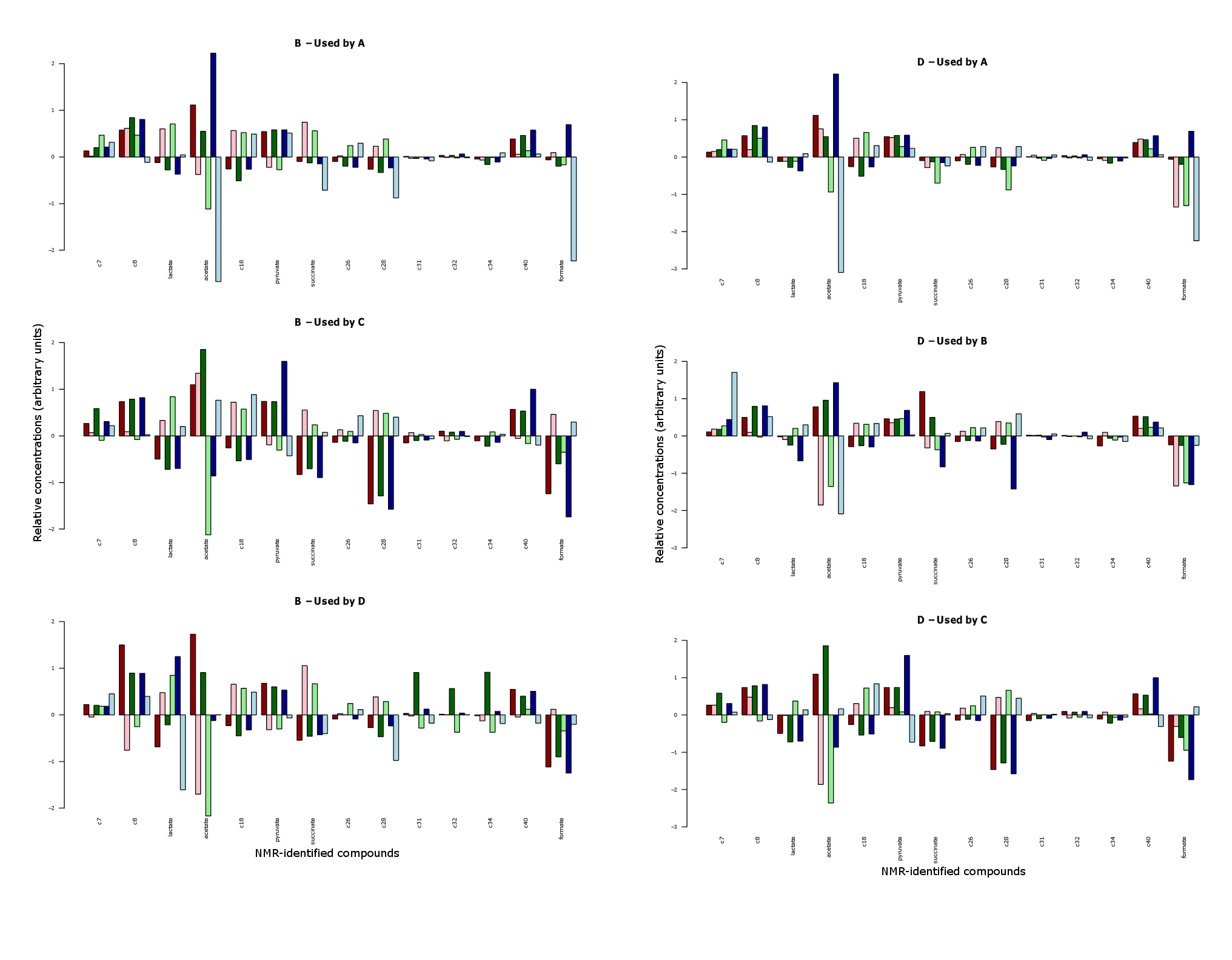

Supplement: Figure S5 — Changes in substrate composition after use by a first species and then species B or D. The difference in the relative size of NMR peaks between tea used by a first species' ancestral (red), monoculture (green), and polyculture (blue) in turn and the relative size of peaks in unused beech tea; together with the change in the size of the peak after a second species grew on medium already used by the first species (then filter sterilised) for the same treatments (ancestral, pink; monoculture, light green; polyculture, light blue). The order of bars for each compound is first species ancestral, second species ancestral, first species monoculture, second species monoculture, first species polyculture, and second species polyculture. Positive values indicate production of a compound, and negative values indicate consumption of a compound relative to the starting medium. To improve clarity of the figure and focus on compounds of interest for cross-feeding, only compounds in which at least one isolate generated an increase in peak size of 0.5 are shown. Only species B and species D were used as the second species, chosen to represent two species showing different results in the growth assays. Evidence of evolved cross-feeding in polyculture is apparent when high blue peaks (generation of the compound by the first species) are associated with low purple peaks (use of the compound by the second species). For example, the species A polyculture isolate produces formate, which in turn is used up by both species B and D. (TIF) [file pbio.1001330.s005.tif]

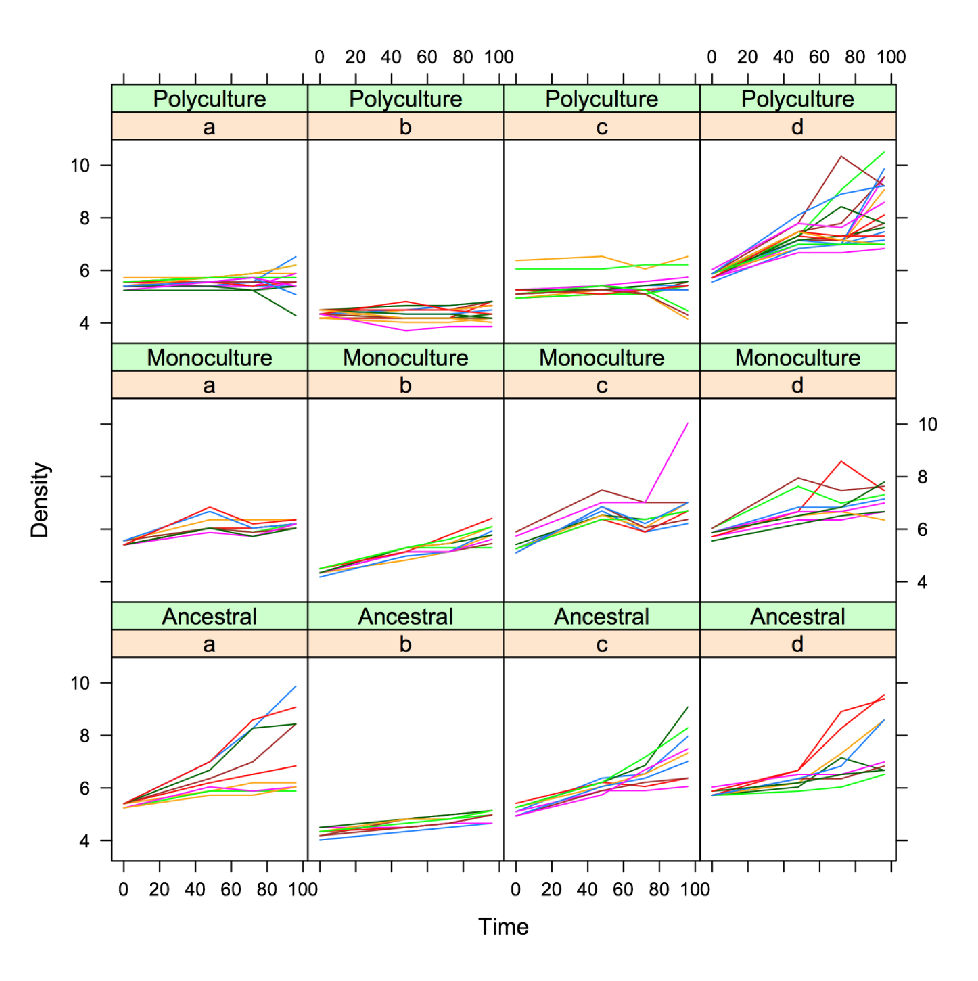

Supplement: Figure S6 — Growth of replicates of each species in assays on unused beech tea across the three treatments. The y-axes are log(cell counts per ml), and x-axes are time since start in hours. Ancestral isolates of all four species grew linearly over the assay period on unused beech tea (ANOVA comparing a model with time as a factor versus a model with time as a continuous variable, likelihood ratio = 6.9, df = 13 and 21, p = 0.55). The monoculture isolates displayed significantly non-linear growth (ANOVA comparing models with time as a factor and as a continuous variable, L-ratio 39.3, p<0.0001). In species A, B, and C there was a reduction in growth rate between day 2 and 3 followed by recovery by day 4. In species D, there was a successive decline in growth rate. In each case, growth between day 0 and day 2 was faster than at any later period. Polyculture isolates grew linearly over the assay period (ANOVA comparing models with time as a factor and as a continuous variable, L-ratio 27.7, p<0.001). (TIF) [file pbio.1001330.s006.tif]

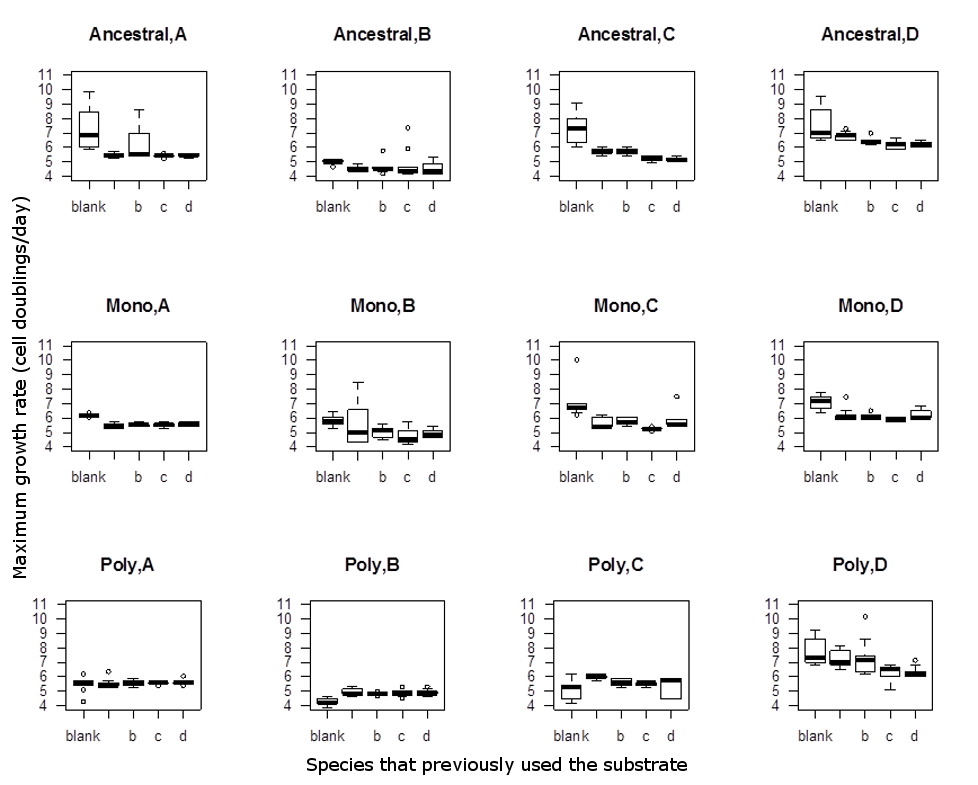

Supplement: Figure S7 — Boxplots of the density after 4 d (log10) across species and substrates. The dark line shows the median, the box limits show the inter-quartile range, and whiskers/points indicate extreme values. Key findings based on comparing Vmax remain the same when comparing amount of growth by day 4: species A grows well on unused tea in ancestral and monoculture treatments, but not when it has evolved in polyculture. Species B and C shift from having reduced growth on used tea in ancestral and monoculture isolates to having enhanced growth in polyculture treatments. Species D evolves to have stronger negative effects of used tea in monoculture than in ancestral isolates, but evolves even better growth on unused tea when it evolves in polycultures than in either ancestral or monoculture isolates. (TIF) [file pbio.1001330.s007.tif]

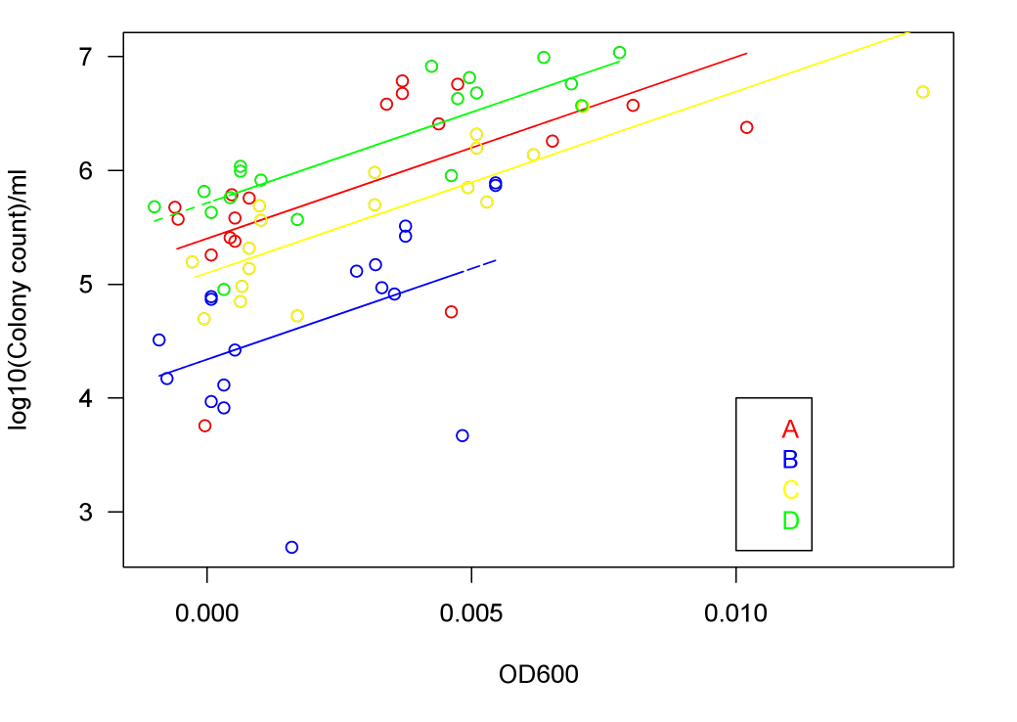

Supplement: Figure S8 — Scatter plot showing the linear relationship between OD600 and log colony counts. The model simplified to retain species and OD600, but no interaction terms (i.e., different intercept for calibration line for each species, but same slopes, F 4,67 = 32.9, p<0.0001, r 2 = 0.64). The fitted lines were used to calibrate in units of log(number of cells) per ml. (TIF) [file pbio.1001330.s008.tif]
