# Supplementary material for: Species Interactions Alter Evolutionary Responses to a Novel Environment
Source: PLoS Biol. 2012 May 15;10(5):e1001330. doi: 10.1371/journal.pbio.1001330 (PMC3352820; doi:10.1371/journal.pbio.1001330)
Supplement: Table S1 — Molecular identification of bacterial isolates. (DOCX) [file pbio.1001330.s009.docx]

Table S1. **Molecular identification of bacterial isolates.**

| Species | 16S rDNA sequence | Closest BLAST hits on NCBI | Closest match on Ribosomal Database Project II database | Likely trophic type |
| --- | --- | --- | --- | --- |
| A | TACCGTACATTCAGCTTCTCACACGTGAAAAGGTTTATTCCGGTACAAAAGCAGTTTACAACCCGTAGGGCCGTCTTCCTGCACGCGGCATGGCTGGTTCAGGCTTGCGCCCATTGACCTAATATTCCTTACTGCTGCCTCCCGTAGGCCCCCCGTGCCCCCGCCCCGCCCGCCGCGCGCGGCGGGCGGGGC | 10 uncultured bacteria clones (99% identity)  Sphingobacteriaceae bacterium (99% identity)  *Pedobacter sp.* (99% identity) | Unclassified Sphingobacteriaceae | Aerobic heterotrophy |
| B | ACGTCATGTTCAGTGCTATTAACACTTAACCCTTCCTCCTCGCTGAAAGTGCTTTACAACCCGAAGGCCTTCTTCACACACGCGGCATGGCTGCATCAGGCTTGCGCCCATTGTGCAATATTCCCCACTGCTGCCTCCCGTAGGCCC | *Yersinia ruckeri* (100% identity)  26 *Yersinia ruckeri* (99% identity)  Uncultured proteobacterium (99% identity)  *Serratia proteomaculans* (99% identity)  *Yersinia ruckeri* (97% identity)  *Rahnella* sp. (97% identity) | Unclassified Enterobacteriaceae | Aerobic heterotrophy |
| C | TACGTCAAACAGCAAAGTATTAATTTACTGCCCTTCCTCCCAACTTAAAGTGCTTTACAATCCGAAGACCTTCTTCACACACGCGGCATGGCTGGATCAGGCTTTCGCCCATTGTCCAATATTCCCCACTGCTGCCTCCCGTAGGCCCCCCGTGCCCCCGCCCCGCC | 10 *Pseudomonas* sp. (100% identity)  2 uncultured bacteria clones (100% identity)  15 *Pseudomonas* sp (99% identity) | Unclassified Pseudomonaceae | Aerobic heterotrophy |
| D | TACTGTCATTATCATCCCTGGTAAAAGAGCTTTACAACCCTAAGGCCTTCATCACTCACGCGGCATTGCTGGATCAGGGTTGCCCCCATTGTCCAATATTCCCCACTGCTGCCTCCCGTAGGCCCCCCGTGCCCCCGCCCCGCCCGCCGCGCGCGGCGGG | 4 uncultured bacteria clones (100% identity)  *Sphingomonas sp.* (100% identity)  *Novosphingobium* sp. (100% identity)  7 uncultured bacteria clones (100% identity) | Unclassified Alphaproteobacteria | Aerobic heterotrophy |
| E | GTACCGTCAGCTGATTCACGAATCAGTGTTTCTTCCTGTGCAAAAGCAGTTTACAATCCATAGGACCGTCATCCTGCACGCGGCATGGCTGGTTCAGGCTTGCGCCCATTGACCAATATTCCTCACTGCTGCCTCCCGTAGGCCCCCCGTGCCCCCGCCCCG | Uncultured Bacteriodetes bacterium clone (100% identity)  Uncultured bacterium clone (99% identity)  2 *Flavobacterium sp*. (99% identity)  4 uncultured bacteria clone (99% identity)  3 *Flavobacterium sp*. (97% identity) | Unclassified Flavobacteriales | Aerobic heterotrophy |
