# Supplementary material for: Species Interactions Alter Evolutionary Responses to a Novel Environment
Source: PLoS Biol. 2012 May 15;10(5):e1001330. doi: 10.1371/journal.pbio.1001330 (PMC3352820; doi:10.1371/journal.pbio.1001330)
Supplement: Table S2 — Description and photographs of growth morphology of each species on agar plates. (DOCX) [file pbio.1001330.s010.docx]

Table S2**. Description and photographs of growth morphology of each species on agar plates.**

| **Species A**  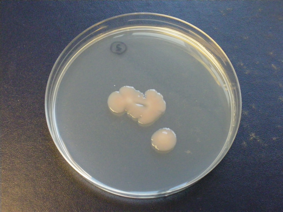  Pink colonies with a glossy, smooth surface. | **Species B**  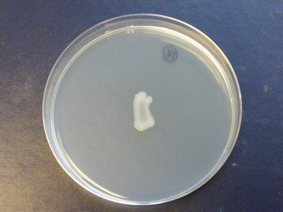  Opaque white colonies with an irregular edge. | **Species C**  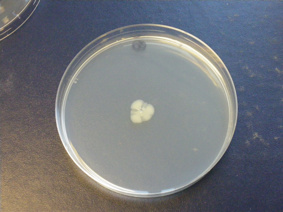  Translucent white colonies with a smooth edge. |
| --- | --- | --- |
| **Species D**  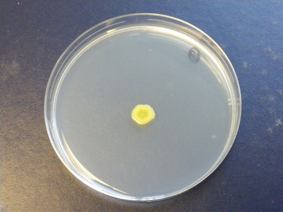  Opaque yellow colonies with a glossy surface. | **Species E**  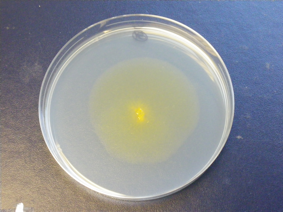  Yellow colonies, opaque centre, translucent at edge. |  |
