# Supplementary material for: Species Interactions Alter Evolutionary Responses to a Novel Environment
Source: PLoS Biol. 2012 May 15;10(5):e1001330. doi: 10.1371/journal.pbio.1001330 (PMC3352820; doi:10.1371/journal.pbio.1001330)
Supplement: Table S3 — Densities, doubling rates, and effective population sizes of each species during the evolution experiments. (DOCX) [file pbio.1001330.s011.docx]

Table S3. **Densities, doubling rates and effective population sizes of each species during the evolution experiments.**

| Treatment | Species | *N_0_* | *N_t_* | No. cell doublings per day | Total number of generations | Effective population size |
| --- | --- | --- | --- | --- | --- | --- |
| Monoculture | A | 6.23 ± 0.04 | 7.51 ± 0.04 | 1.21 ± 0.07 | 67.7 ± 4 | 6.99 ± 0.02 |
|  | B | 5.34 ± 0.21 | 6.64 ± 0.21 | 1.19 ± 0.34 | 66.6 ± 18.8 | 5.97 ± 0.17 |
|  | C | 5.56 ± 0.09 | 6.71 ± 0.13 | 1.09 ± 0.09 | 60.9 ± 5 | 6.27 ± 0.1 |
|  | D | 6.13 ± 0.03 | 7.45 ± 0.04 | 1.25 ± 0.08 | 70.2 ± 4.8 | 6.91 ± 0.03 |
|  | E | 4.14 ± 0.35 | 5.21 ± 0.36 | 1.09 ± 0.3 | 61.2 ± 17 | 4.53 ± 0.33 |
| Polyculture | A | 4.94 ± 0.05 | 6.27 ± 0.07 | 1.27 ± 0.12 | 71.1 ± 6.9 | 5.72 ± 0.04 |
|  | B | 5.04 ± 0.18 | 6.58 ± 0.2 | 1.47 ± 0.13 | 82.2 ± 7.3 | 5.89 ± 0.18 |
|  | C | 5.53 ± 0.06 | 6.73 ± 0.09 | 1.14 ± 0.1 | 63.6 ± 5.4 | 6.26 ± 0.06 |
|  | D | 5.35 ± 0.06 | 6.62 ± 0.06 | 1.2 ± 0.08 | 67 ± 4.5 | 6.11 ± 0.04 |
|  | E | 4.34 ± 0.13 | 5.71 ± 0.12 | 1.37 ± 0.29 | 76.6 ± 16.1 | 5.08 ± 0.05 |

Cell densities just before serial transfer events, *N_t_* were estimated from colony counts of samples plated on R2A agar during the course of the experiment. Following Wahl and Gerrish (2001) [5], we calculated the average effective population size during the experiment as *N_0_log(2)t*, where *N_0_* is the starting cell count in the tube after a transfer event, which is given by our dilution factor (0.05) times *N_t_* prior to transfer, and *t* is the estimated number of cell doublings between transfer events. Standard errors are shown for estimates. Cell densities were significantly lower on average in the polycultures than in monocultures in species A and D (ANOVA, both F_1,16_>18.1, p<0.0001): the other species showed no significant difference although species B and E followed the same trend.
