# Supplementary material for: Species Interactions Alter Evolutionary Responses to a Novel Environment
Source: PLoS Biol. 2012 May 15;10(5):e1001330. doi: 10.1371/journal.pbio.1001330 (PMC3352820; doi:10.1371/journal.pbio.1001330)
Supplement: Table S4 — Linear mixed effects model comparisons. (DOCX) [file pbio.1001330.s012.docx]

Table S4. **Linear mixed effects model comparisons.**

| Models compared | Likelihood ratio | Degrees of freedom | p |
| --- | --- | --- | --- |
| 1) Model 1: species X treatment X substrate X time  2) Model 1 but removing the highest order interaction term* | 585.9 | 217, 325 | <0.0001 |
| 1) Model 1  2) Model 2: species X treatment X interaction X time # | 449.9 | 197, 325 | <0.0001 |

* Comparison shows that all explanatory variables remain in model including the four-way interaction term

# Comparison shows that there are differences in interaction strength between different combinations of species: substrate (= used by species a, b, c or d) is a better explanatory variable than interaction (=unused, used by own species, used by a different species).
