# Supplementary material for: Species Interactions Alter Evolutionary Responses to a Novel Environment
Source: PLoS Biol. 2012 May 15;10(5):e1001330. doi: 10.1371/journal.pbio.1001330 (PMC3352820; doi:10.1371/journal.pbio.1001330)
Supplement: Text S1 — Additional methods and references. (DOC) [file pbio.1001330.s013.doc]

**SUPPORTING INFORMATION**

Supporting text S1

Tables S1 to S4

Figure Legends

Figures S1 to S8

**Supporting Text S1 – additional methods**

*Isolating bacteria*

To ensure that starting species comprised single genotypes, single colonies from R2A agar plates spread with tree-hole water were re-suspended in liquid medium and then re-plated on R2A agar, before picking off final single colony isolates for storage in glycerol at -84ºC. Isolates were identified from 16S rDNA sequence using primers 27F [1] and 534R [2].

*Evolution experiments*

Cultures were established in 30ml universal tubes containing 2ml of beech tea and inoculated initially with 50µl from established cultures of bacteria in beech tea. In ‘monoculture’ treatments, cultures were started with 50µl of each species in turn cultured in 2ml of medium in isolation. In ‘polyculture’ treatments, inoculates of 10µl of each of the species was added to the same tube to create a five-species community of bacteria. The optical densities at 600nm (OD_600_) were measured for all of the stock cultures before inoculation to ensure that starting densities were similar for all species.

*Linear mixed effects models*

For each analysis, we first fitted the most complex model with all interactions among the explanatory variables as fixed effects, and time nested within a grouping factor specifying each assay well as random effects. Visual inspection of residuals against fitted values and of quantile normal plots were used to judge model assumptions [3]. Following statistical comparison of alternative models of variance-covariance structure, density was included as a variance covariate in all models [4]. To test for non-linear growth over time, we repeated the procedure both assuming time to be continuous and as a factor (four levels), and used ANOVA to compare models fitted using the maximum likelihood criterion. Finally, we attempted to simplify the maximum model by removing highest order interaction terms and using ANOVA to compare nested models fitted using maximum likelihood. The minimum adequate model was refitted using restricted maximum likelihood (REML) for reporting. For assays on used beech tea, we compared models with either ‘interaction type’ (unused, used by intraspecific isolate, or used by interspecific isolate) or ‘substrate’ (unused, used by species A, used by species B etc.) to test for significant differences in interaction strengths among species. We used growth over the first 48 hours to summarize changes in maximum growth rate, *Vmax*: in all assays, growth was either linear or fastest during the first 48 hours (Fig. S6). Similar conclusions about the evolutionary effects of species interactions were reached using density at 96 hours as a measure of carrying capacity instead of focusing on initial growth rate (Fig. S7).

References:

1. Lane DJ (1991) in *16S/23S rRNA sequencing. Nucleic acid techniques in bacterial systematics,* eds Stackebrandt E, Goodfellow M (Wiley, New York), pp 115–147.
2. Muyzer G, Dewaal EC, Uitterlinden AG (1993) Profiling of complex microbial-populations by denaturing gradient gel-electrophoresis analysis of polymerase chain reaction-amplified genes-coding for 16S ribosomal-RNA. *Appl Environ Microbiol* 59: 695–700.
3. Crawley MJ (2007) in *The R Book*. (Wiley, New York).
4. Pinheiro JC, Bates DM (2000) in *"Mixed-Effects Models in S and S-PLU*S". (Springer), p 274: 1620 1799-1805.
5. Wahl LM, Gerrish PJ (2001) The probability that beneficial mutations are lost in populations with periodic bottlenecks. Evolution 55: 2606-2610.

Table S1. **Molecular identification of bacterial isolates.**

| Species | 16S rDNA sequence | Closest BLAST hits on NCBI | Closest match on Ribosomal Database Project II database | Likely trophic type |
| --- | --- | --- | --- | --- |
| A | TACCGTACATTCAGCTTCTCACACGTGAAAAGGTTTATTCCGGTACAAAAGCAGTTTACAACCCGTAGGGCCGTCTTCCTGCACGCGGCATGGCTGGTTCAGGCTTGCGCCCATTGACCTAATATTCCTTACTGCTGCCTCCCGTAGGCCCCCCGTGCCCCCGCCCCGCCCGCCGCGCGCGGCGGGCGGGGC | 10 uncultured bacteria clones (99% identity)  Sphingobacteriaceae bacterium (99% identity)  *Pedobacter sp.* (99% identity) | Unclassified Sphingobacteriaceae | Aerobic heterotrophy |
| B | ACGTCATGTTCAGTGCTATTAACACTTAACCCTTCCTCCTCGCTGAAAGTGCTTTACAACCCGAAGGCCTTCTTCACACACGCGGCATGGCTGCATCAGGCTTGCGCCCATTGTGCAATATTCCCCACTGCTGCCTCCCGTAGGCCC | *Yersinia ruckeri* (100% identity)  26 *Yersinia ruckeri* (99% identity)  Uncultured proteobacterium (99% identity)  *Serratia proteomaculans* (99% identity)  *Yersinia ruckeri* (97% identity)  *Rahnella* sp. (97% identity) | Unclassified Enterobacteriaceae | Aerobic heterotrophy |
| C | TACGTCAAACAGCAAAGTATTAATTTACTGCCCTTCCTCCCAACTTAAAGTGCTTTACAATCCGAAGACCTTCTTCACACACGCGGCATGGCTGGATCAGGCTTTCGCCCATTGTCCAATATTCCCCACTGCTGCCTCCCGTAGGCCCCCCGTGCCCCCGCCCCGCC | 10 *Pseudomonas* sp. (100% identity)  2 uncultured bacteria clones (100% identity)  15 *Pseudomonas* sp (99% identity) | Unclassified Pseudomonaceae | Aerobic heterotrophy |
| D | TACTGTCATTATCATCCCTGGTAAAAGAGCTTTACAACCCTAAGGCCTTCATCACTCACGCGGCATTGCTGGATCAGGGTTGCCCCCATTGTCCAATATTCCCCACTGCTGCCTCCCGTAGGCCCCCCGTGCCCCCGCCCCGCCCGCCGCGCGCGGCGGG | 4 uncultured bacteria clones (100% identity)  *Sphingomonas sp.* (100% identity)  *Novosphingobium* sp. (100% identity)  7 uncultured bacteria clones (100% identity) | Unclassified Alphaproteobacteria | Aerobic heterotrophy |
| E | GTACCGTCAGCTGATTCACGAATCAGTGTTTCTTCCTGTGCAAAAGCAGTTTACAATCCATAGGACCGTCATCCTGCACGCGGCATGGCTGGTTCAGGCTTGCGCCCATTGACCAATATTCCTCACTGCTGCCTCCCGTAGGCCCCCCGTGCCCCCGCCCCG | Uncultured Bacteriodetes bacterium clone (100% identity)  Uncultured bacterium clone (99% identity)  2 *Flavobacterium sp*. (99% identity)  4 uncultured bacteria clone (99% identity)  3 *Flavobacterium sp*. (97% identity) | Unclassified Flavobacteriales | Aerobic heterotrophy |

Table S2**. Description and photographs of growth morphology of each species on agar plates.**

| **Species A**  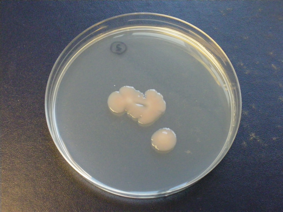  Pink colonies with a glossy, smooth surface. | **Species B**  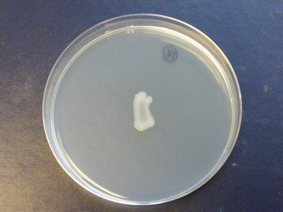  Opaque white colonies with an irregular edge. | **Species C**  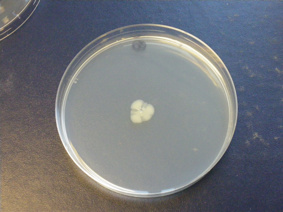  Translucent white colonies with a smooth edge. |
| --- | --- | --- |
| **Species D**  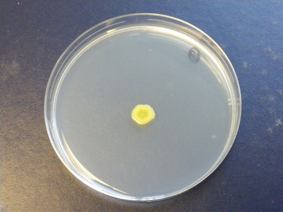  Opaque yellow colonies with a glossy surface. | **Species E**  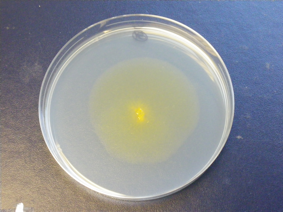  Yellow colonies, opaque centre, translucent at edge. |  |

Table S3. **Densities, doubling rates and effective population sizes of each species during the evolution experiments.**

| Treatment | Species | *N_0_* | *N_t_* | No. cell doublings per day | Total number of generations | Effective population size |
| --- | --- | --- | --- | --- | --- | --- |
| Monoculture | A | 6.23 ± 0.04 | 7.51 ± 0.04 | 1.21 ± 0.07 | 67.7 ± 4 | 6.99 ± 0.02 |
|  | B | 5.34 ± 0.21 | 6.64 ± 0.21 | 1.19 ± 0.34 | 66.6 ± 18.8 | 5.97 ± 0.17 |
|  | C | 5.56 ± 0.09 | 6.71 ± 0.13 | 1.09 ± 0.09 | 60.9 ± 5 | 6.27 ± 0.1 |
|  | D | 6.13 ± 0.03 | 7.45 ± 0.04 | 1.25 ± 0.08 | 70.2 ± 4.8 | 6.91 ± 0.03 |
|  | E | 4.14 ± 0.35 | 5.21 ± 0.36 | 1.09 ± 0.3 | 61.2 ± 17 | 4.53 ± 0.33 |
| Polyculture | A | 4.94 ± 0.05 | 6.27 ± 0.07 | 1.27 ± 0.12 | 71.1 ± 6.9 | 5.72 ± 0.04 |
|  | B | 5.04 ± 0.18 | 6.58 ± 0.2 | 1.47 ± 0.13 | 82.2 ± 7.3 | 5.89 ± 0.18 |
|  | C | 5.53 ± 0.06 | 6.73 ± 0.09 | 1.14 ± 0.1 | 63.6 ± 5.4 | 6.26 ± 0.06 |
|  | D | 5.35 ± 0.06 | 6.62 ± 0.06 | 1.2 ± 0.08 | 67 ± 4.5 | 6.11 ± 0.04 |
|  | E | 4.34 ± 0.13 | 5.71 ± 0.12 | 1.37 ± 0.29 | 76.6 ± 16.1 | 5.08 ± 0.05 |

Cell densities just before serial transfer events, *N_t_* were estimated from colony counts of samples plated on R2A agar during the course of the experiment. Following Wahl and Gerrish (2001) [5], we calculated the average effective population size during the experiment as *N_0_log(2)t*, where *N_0_* is the starting cell count in the tube after a transfer event, which is given by our dilution factor (0.05) times *N_t_* prior to transfer, and *t* is the estimated number of cell doublings between transfer events. Standard errors are shown for estimates. Cell densities were significantly lower on average in the polycultures than in monocultures in species A and D (ANOVA, both F_1,16_>18.1, p<0.0001): the other species showed no significant difference although species B and E followed the same trend.

**Table S4. Linear mixed effects model comparisons.**

| Models compared | Likelihood ratio | Degrees of freedom | p |
| --- | --- | --- | --- |
| 1) Model 1: species X treatment X substrate X time  2) Model 1 but removing the highest order interaction term* | 585.9 | 217, 325 | <0.0001 |
| 1) Model 1  2) Model 2: species X treatment X interaction X time # | 449.9 | 197, 325 | <0.0001 |

* Comparison shows that all explanatory variables remain in model including the four-way interaction term

# Comparison shows that there are differences in interaction strength between different combinations of species: substrate (= used by species a, b, c or d) is a better explanatory variable than interaction (=unused, used by own species, used by a different species).

**Supplementary Figure Legends**

Figure S1. **Maximum growth rates for each species and evolution treatment when grown in ‘used’ and ‘unused’ substrate.** Boxplots of maximum growth rates, *V_MAX_*, in cell doublings per day across evolution treatments, species and substrates. The dark line shows the median, the box limits show the inter-quartile range, and whiskers/points indicate extreme values.

Figure S2. **Amounts of compounds identified from distinct peaks in the NMR spectrum of unused beech tea.** Bars show the size of the major peak for each distinct compound relative to the size of the standard, DSS; hence peak heights are dimensionless. The location of each peak on the spectrum is shown after each name (peak shift in parts per million).

Figure S3. **NMR peaks for each species and treatment.** The difference in the size of NMR peaks between tea used by ancestral (dark grey), monoculture (mid grey) and polyculture (light grey) in turn and the size of peaks in unused beech tea. Positive values indicate production of a compound and negative values indicate consumption of a compound. Peak sizes are expressed relative to the size of the standard, DSS, and hence are dimensionless.

Figure S4. **Contribution of each compound to variation between treatments.** Loadings of the first four principal components of resource use and production of the four surviving species across ancestral, monoculture and polyculture treatments. The input data were the difference between the size of the peak in medium used by the isolate and the size of the peak in the beech tea (i.e. the data in Fig. S3). Bars indicate the correlation coefficient between variation in each compound and the relevant principal component. The percentage of total variation described by each principal component is shown above each plot; together they explain 90.1% of the total variation.

Figure S5. **Changes in substrate composition after use by a first species and then species B or D.** The difference in the relative size of NMR peaks between tea used by a first species’ ancestral (red), monoculture (green) and polyculture (blue) in turn and the relative size of peaks in unused beech tea; together with the change in the size of the peak after a second species grew on medium already used by the first species (then filter sterilised) for the same treatments (ancestral = pink, monoculture = light green, polyculture = light blue). The order of bars for each compound is first species ancestral, second species ancestral, first species monoculture, second species monoculture, first species polyculture, second species polyculture. Positive values indicate production of a compound and negative values indicate consumption of a compound relative to the starting medium. To improve clarity of the figure and focus on compounds of interest for cross-feeding, only compounds in which at least one isolate generated an increase in peak size of 0.5 are shown. Only species B and species D were used as the second species, chosen to represent two species showing different results in the growth assays. Evidence of evolved cross-feeding in polyculture is apparent when high blue peaks (generation of the compound by the first species) are associated with low purple peaks (use of the compound by the second species). For example, the species A polyculture isolate produces formate, which in turn is used up by both species B and D.

Figure S6. **Growth of replicates of each species in assays on unused beech tea across the three treatments.** Y-axes are log(cell counts per ml), and x-axes are time since start in hours. Ancestral isolates of all four species grew linearly over the assay period on unused beech tea (ANOVA comparing a model with time as a factor versus a model with time as a continuous variable, likelihood ratio = 6.9, df = 13 and 21, p=0.55). The monoculture isolates displayed significantly non-linear growth (ANOVA comparing models with time as a factor and as a continuous variable, L-ratio 39.3, p<0.0001). In species A, B, and C there was a reduction in growth rate between day 2 and 3 followed by recovery by day 4. In species D, there was a successive decline in growth rate. In each case, growth between day 0 and day 2 was faster than at any later period. Polyculture isolates grew linearly over the assay period (ANOVA comparing models with time as a factor and as a continuous variable, L-ratio 27.7, p<0.001).

Figure S7. **Boxplots of the density after 4 days (log10) across species and substrates.** The dark line shows the median, the box limits show the inter-quartile range, and whiskers/points indicate extreme values. Key findings based on comparing Vmax remain the same when comparing amount of growth by day 4: species A grows well on unused tea in ancestral and monoculture treatments, but not when it has evolved in polyculture. Species B and C shift from having reduced growth on used tea in ancestral and monoculture isolates to having enhanced growth in polyculture treatments. Species D evolves to have stronger negative effects of used tea in monoculture than in ancestral isolates, but evolves even better growth on unused tea when it evolves in polycultures than in either ancestral or monoculture isolates.

Figure S8. **Scatter plot showing the linear relationship between OD_600_ and log colony counts.** The model simplified to retain species and OD_600_, but no interaction terms (i.e. different intercept for calibration line for each species, but same slopes, F_4,67_=32.9, p<0.0001, r^2^ = 0.64). The fitted lines were used to calibrate in units of log(number of cells) per ml.
